# NMR based SARS-CoV-2 Antibody Screening

**DOI:** 10.1021/jacs.1c03945

**Published:** 2021-05-21

**Authors:** Marta V. Schoenle, Yang Li, Meng Yuan, Michael W. Clarkson, Ian A. Wilson, Wolfgang Peti, Rebecca Page

**Affiliations:** 1 Biochemistry Graduate Program, University of Arizona, Tucson, AZ 85721, USA; 2 Department of Molecular Biology and Biophysics, University of Connecticut Health Center, Farmington, CT 06030, USA; 3 Department of Integrative Structural and Computational Biology, The Scripps Research Institute, La Jolla, CA 92037, USA; 4 Deparment of Chemistry and Biochemistry, University of Arizona, Tucson, AZ 85721 USA; 5 The Skaggs Institute for Chemical Biology, The Scripps Research Institute, La Jolla, CA, 92037, USA; 6 Department of Cell Biology, University of Connecticut Health Center, Farmington, CT 06030, USA

## Abstract

The severe acute respiratory syndrome coronavirus 2 (SARS-CoV2) entry into cells is a complex process that involves (1) recognition of the host entry receptor, angiotensin converting enzyme 2 (ACE2), by the SARS-CoV-2 spike protein receptor binding domain (RBD) and (2) the subsequent fusion of the viral and cell membranes. Our long-term immune-defense is the production of antibodies (Abs) that recognize the SARS-CoV2 RBD and successfully block viral infection. Thus, to understand immunity against SARS-CoV-2, a comprehensive molecular understanding of how human SARS-CoV-2 Abs recognize the RBD is needed. Here, we report the sequence specific backbone assignment of the SARS-CoV-2 RBD and, furthermore, demonstrate that biomolecular NMR spectroscopy chemical shift perturbation (CSP) mapping successfully and rapidly identifies the molecular epitope of RBD-specific mAbs. By incorporating NMR-based CSP mapping with other molecular techniques to define RBD-mAb interactions and then correlating these data with neutralization efficacy, new approaches for developing improved vaccines and COVID19 mAb-based therapies will be greatly accelerated.

## Graphical Abstract

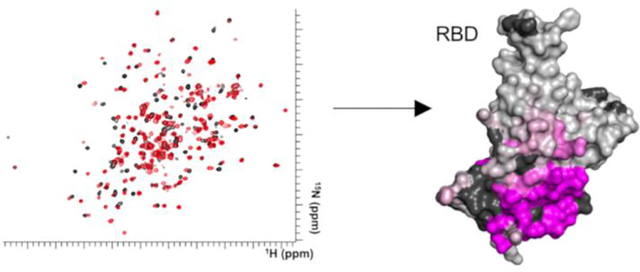

Coronavirus disease 2019 (COVID-19) is an ongoing global pandemic caused by the severe acute respiratory syndrome coronavirus-2 (SARS-Cov-2) ssRNA virus. Although a number of vaccines are now available to prevent serious disease and hospitalization^1^, additional treatment options, such as antivirals, anti-inflammatories, and monoclonal antibody (mAb) therapies are desperately needed for critically ill patients battling the infection. Further, the emergence of escape variants portend the need for new or modified vaccines to combat novel variants^2–4^.

It is now well-established that many neutralizing antibodies isolated from convalescent patients target the SARS-CoV-2 spike, or ‘S’, protein^5,6^. The S protein is a transmembrane glycoprotein that forms homotrimers on the SARS-CoV-2 surface and mediates host cell entry and fusion via binding the angiotensin-converting enzyme 2 (ACE2)^7–9^. The S protein receptor binding domain (RBD) of the spike protein interacts with angiotensin-converting enzyme 2 (ACE2)^9^. Of the 2076 SARS-CoV-2 targeting mAbs isolated since May 2020, 55% (1150) target the RBD domain. Furthermore, 51% of these (585) are reported as neutralizing, demonstrating the central importance of RBD for eliciting potent and effective immune responses^10^.

Defining the molecular basis of mAb recognition of the RBD provide key insights into antigenicity and sites of vulnerability, providing key molecular information to improve existing COVID19 therapies and vaccines^11–14^. To date, 116 RBD-mAb structures have been determined using either X-ray crystallography and/or cryoelectron microscopy (cryo-EM)^10^. These data have led to the discovery of three key, distinct RBD epitopes: the receptor binding site (RBS; with 4 subepitopes), the CR3022 binding site and the S309 binding site (Figs. 1A, S1, green, orange and lavender surfaces, respectively; CR3022 and S309 are RBD-specific mAbs)^12,15,16^. However, if and how these major RBD epitopes are subdivided into distinct classes of sub-epitopes that correlate with neutralization efficacy is an important, actively researched question. This is because the 116 mAb:RBD structures represent only a fraction (10%) of the isolated mAbs demonstrated to bind the RBD. Despite the critical insights into the mechanisms of SARS-CoV-2 neutralization provided by these structures, an additional complementary experimental method that is fast and provides molecular data regarding mAb-RBD binding mechanism would greatly augment and accelerate these efforts.

Here, we show that biomolecular NMR chemical shift perturbation (CSP) mapping using (^2^H,^15^N)-labeled RBD titrated with RBD-binding Fab fragments (antibody binding fragment of mAb) can help fill this missing knowledge gap. Our data demonstrate that despite the size of the Fab-RBD complex (~70 kDa), the 2D [^1^H,^15^N] TROSY spectra of the RBD:RBD-binding Fab complex are of outstanding quality, allowing the CSPs to be readily identified and the RBD residues that mediate mAb binding to be identified. Further, because it is a solution-based technique, multiple mapping experiments can be performed in a timely manner, limited only by the time it takes to express and purify the Fabs themselves. By using NMR CSP mapping in addition to X-ray crystallography and cryo-EM, the full catalog of RBD epitopes may be molecularly defined, and not just those amenable to crystallographic and/or cryo-EM methodologies. This approach will allow the full collection of epitope-binding mAbs to be classified based on their RBD-binding interface^12,15,16^. Further, correlating neutralization efficacy with each RBD-binding class, as has been initiated for those binding classes identified by crystallography and cryoEM^16^, will lead to novel approaches for developing improved vaccines and COVID19 mAb-based therapies.

Fab:RBD interactions are typically strong (K_D_, nM range)^11^. Thus, these complexes will behave akin to a ~70 kDa protein, which requires strict protein deuteration and the use of transverse relaxation optimized spectroscopy (TROSY)^17^. Uniformly (^2^H,^15^N)- or (^2^H,^15^N,^13^C)-labeled SARS-CoV2 RBD (aa 331–528) was expressed as inclusion bodies in *E. coli* and the protein refolded by rapid dilution using a reduced-oxidized glutathione (GSH:GSSG) redox buffer (Fig. 1B, inset). Properly folded RBD was isolated using size exclusion chromatography and the thermal stability, T_M_, determined to be 49.6 ± 0.1 °C (SEM; n=4).

Because CSP mapping experiments are only capable of defining protein:protein interactions with a highly complete specific backbone assignment of the protein (or proteins) under investigation, we next determined the sequence-specific backbone assignment of the RBD. The fully annotated 2D [^1^H,^15^N] TROSY-spectrum of the RBD is shown in Fig. 1B. The completeness of the sequence-specific backbone assignments of the expected backbone peaks is 88% (166/186). Most residues in functionally critical regions, including the major RBD epitopes, are assigned, with the assignments for the RBS, CR3022 and S309 being 86%, 79% and 89% complete, respectively. Unassigned RBD residues are largely localized to a single patch near the RBD N-terminus, which overlaps just slightly with the bottom tips of the CR3022 and S309 binding surfaces (Fig. 1C). Chemical shift index calculations derived from Cα and Cβ chemical shifts showed that the secondary structure elements of the RBD in solution map perfectly to those observed in the RBD crystal structure (Fig. 1D; compared to RBD crystal structure in PDBID 6w41^11^).

Dynamics of the RBD domain were analyzed using ^15^N{^1^H}-NOE (hetNOE) measurements, which reports on fast time scale (ps to ns) backbone motions. Three regions in the RBD have residues with hetNOE values <0.7: 368–375 loop, 382–387 loop and 476–489 loop, indicating higher flexibility. Consistent with this result, these loops exhibit higher variability in crystals structures of the RBD (resolutions better than 2.25 Å). While loops 368–375 and 382–387 directly flank the CR3022 binding interface, loop 476–489 defines the ridge bound by the ACE2 receptor and many RBS mAbs (Figs. 2A-C).

Next we asked if CSP mapping would be a viable approach for identifying Fab:RBD interaction surfaces. For this proof-of-principle study, we used the Fab from the well-characterized mAb CR3022 (hereafter, the Fab is referred to as CR3022)^18^. CR3022 was derived from a mAb that was originally isolated from a SARS-CoV convalescent patient^19^. It was later shown to cross-react with the RBD from SARS-CoV-2^20^. Because the structure of CR3022 bound to the SARS-CoV-2 RBD has been determined using X-ray crystallography^11^, the overlap between the interface determined using NMR chemical shift perturbation mapping and x-ray crystallography can be readily assessed.

In this experiment, (^2^H,^15^N)-labeled RBD was incubated with increasing ratios of unlabeled CR3022 (1:1.1, 1:2, 1:2.25) and a 2D [^1^H,^15^N] TROSY spectrum recorded. The spectrum is of excellent quality for a complex of this size (70 kDa) with no apparent loss of signals due to line broadening and the ^1^H,^15^N cross-peaks are well-resolved (Fig. 3A). Thus, the chemical shift perturbations in the RBD caused by CR3022 binding can be readily detected. Direct comparison of the 2D [^1^H,^15^N] TROSY spectra of the free and CR3022-bound RBD revealed CSPs, which allowed 145 peaks to be readily assigned, 19 of which experience CSPs greater than 2σ (Fig. 3B). The remaining ~20 peaks shifted to such a great extent that they could not be confidently assigned. Mapping the residues that experience CSPs onto the surface of the RBD shows that the majority of the most significantly shifted peaks correspond to residues at the RBD-CR3022 interface (Fig. 3C, D). In particular, comparing the residue-specific CSPs with the corresponding buried solvent accessible surface area shows that the major and two minor interaction sites (major: residues 369–392; minor: residues 427–430, 515–519) as identified using X-ray crystallography (Fig. 3D) are also readily identified using NMR CSP mapping (Fig. 3B), with the major interaction site residues, which constitute 78% of the total RBD BSA, corresponding to those residues that experience the largest CSPs. Further, of the 29 residues that constitute the CR3022 interface, 26 experience a CSP that is either greater than the 2σ threshold or shifted to such an extent they could not be confidently assigned (only K386, D427 and D428 had CSP less than the 2σ threshold). These data unequivocally confirm that CSP mapping is an ideal approach for rapidly identifying the RBD residues that bind RBD-specific Fabs.

The data also show that there are also some small differences between the two techniques. For example, K386 experiences the second largest loss of solvent accessible surface area upon CR3022 binding, while its CSP is less than the 2σ threshold. This is because CR3022 binding fully buries the K386 sidechain, which results in a large loss of solvent accessible surface area, while the CSP measure changes in the amide-proton vectors of the residue backbone. Together, these results demonstrate that, for K386, CR3022 binding interacts predominantly with the sidechain versus the K386 backbone. Conversely, three residues (408–410) experience CSPs greater than the 2σ threshold, yet only one of these residues, R408, has solvent accessible surface area buried by CR3022 and the extent is only minimal (10 Å^2^; sidechain). Although the R408 BSA change is minimal, the CSP data demonstrate that this residue defines a minor interaction site. Thus, NMR CSP mapping, in addition to identifying major binding epitopes, also readily identifies even minor interaction sites.

Together, these data demonstrate that current ambitious efforts to correlate RBD-specific mAb binding mechanisms with neutralization efficacy can be greatly accelerated by incorporating NMR CSP mapping, in addition to X-ray crystallography and cryo-EM, for characterizing the Fab-RBD interface at molecular resolution. Although using CSP mapping for identifying Fab-antigen interfaces is not new^21,22^, it has not been widely used, likely due in part to the need for an antigen that is amenable for NMR spectroscopy. Here, we show that the RBD domain is ideal for these studies and that CSP mapping will allow the full complement of RBD-mAb to be rapidly defined. Because NMR readily detects interactions with even mM affinities, any reduction in affinity due to the absence of glycosylation on RBD residue N343 is not expected to negatively impact epitope identification. Finally, by generating variants of the RBD that are present in novel, more infectious SARS-CoV-2 strains, one can also use CSP mapping to identify, at a molecular level, how the Fab-RBD interaction changes as a result of RBD mutations. Together, by integrating biomolecular liquid-state NMR with x-ray crystallography and cryo-EM for defining all RBD-Fab interactions and correlating these data with neutralization efficacy, it is hoped that new approaches for the development of improved vaccines and COVID19 mAb-based therapies will be more rapidly realized.

## Supplementary Material

Supplementary Material

ASSOCIATED CONTENT

Supporting Information

Experimental protocols and supplemental figures.

## Figures and Tables

**Figure 1.**
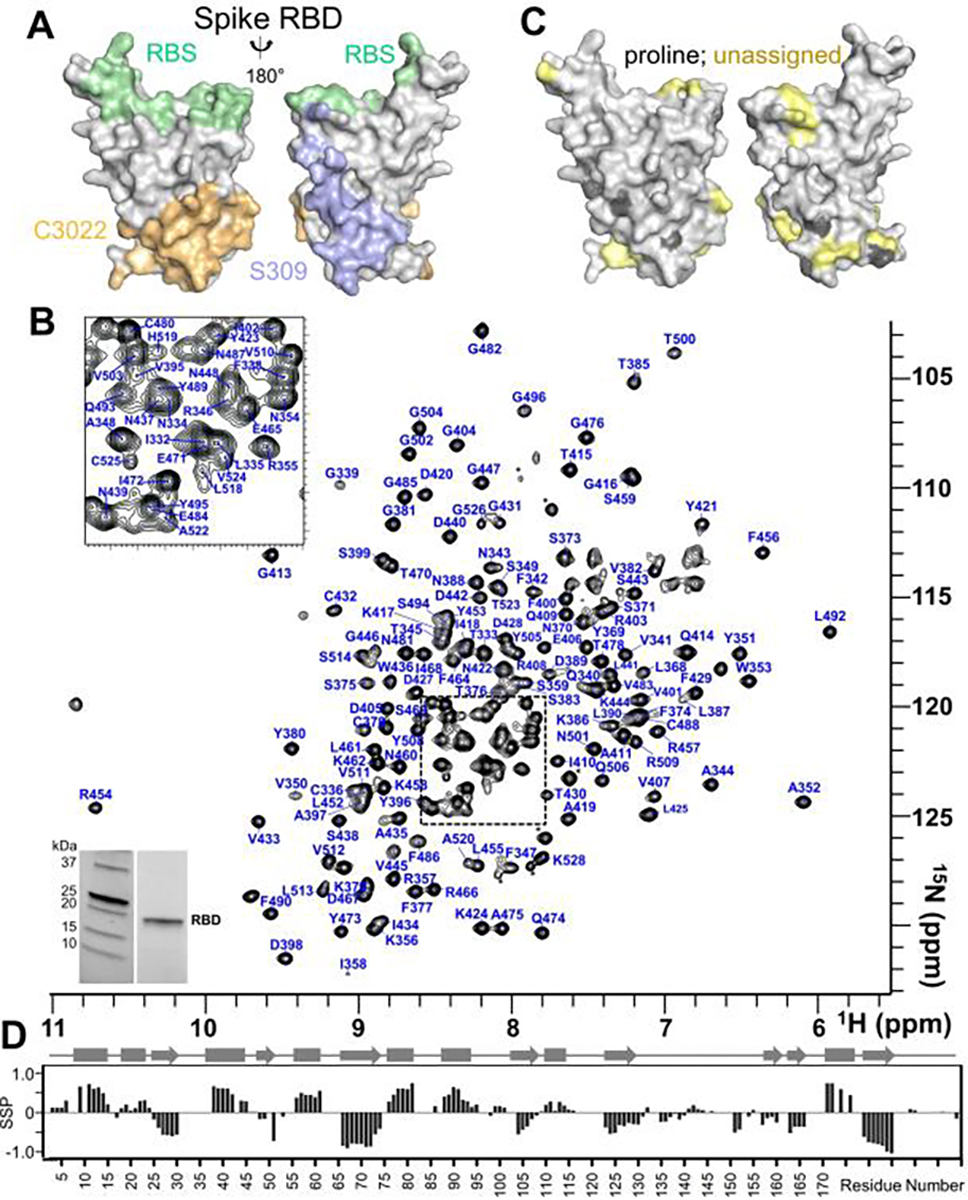
Sequence specific backbone assignment of the SARS-CoV-2 spike receptor binding domain (RBD). **A**. Surface representation of the RBD, with the major known antibody-binding epitopes colored in light green (receptor binding site, RBS), light orange (CR3022 binding site) and lavender (S309 binding site). **B**. Fully annotated 2D [^1^H,^15^N] TROSY spectrum of the RBD (800 MHz ^1^H Larmor). Assigned peaks are labeled with the corresponding residue (single letter code) and number in the protein sequence. **C**. Surface representation of assigned residues. Prolines are shaded dark grey and unassigned residues are shaded light yellow. **D**. Chemical shift index/secondary structure propensity plot of the RBD; secondary structural elements are shown above.

**Figure 2.**
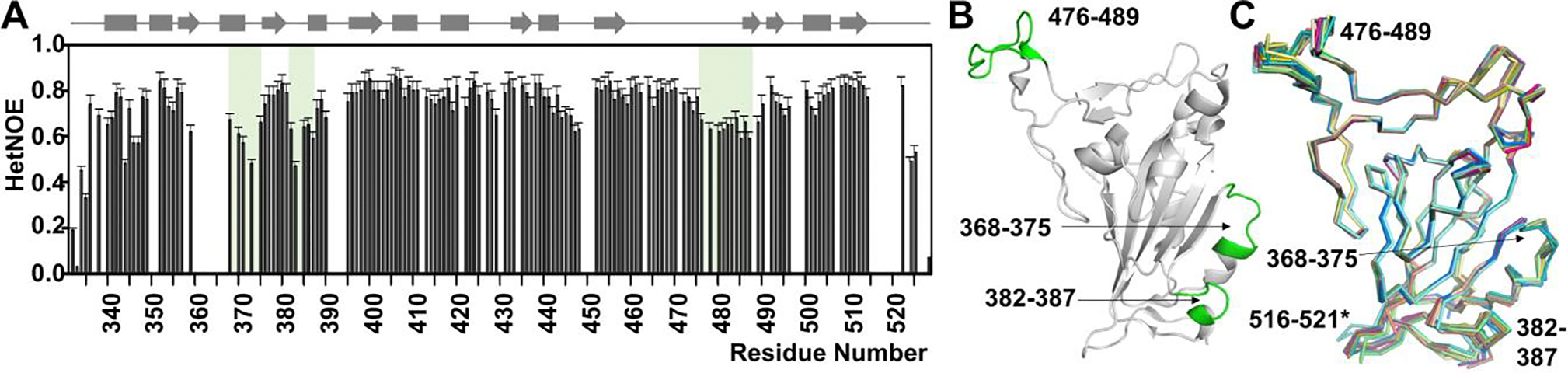
RBD dynamics. **A**. ^15^N{^1^H}-NOE of the RBD (600 MHz ^1^H Larmor) identifies three loops with increased flexibility (light green background: 368–375, 382–387 and 476–489). **B.** Cartoon of the RBD with the three flexible loops colored green; RBD orientation identical to that in Fig. 1A, left panel. **C**. Overlay of RBD crystals structures with the RBD from 6w41 (2.25 Å resolution or better: 7EAN, 7KFY, 7KFX, 7LM8, 7CJF, 7DEU, 7KN5^23^, 7KMI, 7LOP, 7B3O, 6YZ5, 7EAM; average Cα-Cα RMSD is 0.91 Å, 1.18 Å, 0.89 Å, 1.07 Å for loops 368–375, 382–387, 476–489 and 516–521, respectively; overall Cα-Cα RMSD is 0.51 Å). Residues 516–521 (*) are unassigned.

**Figure 3.**
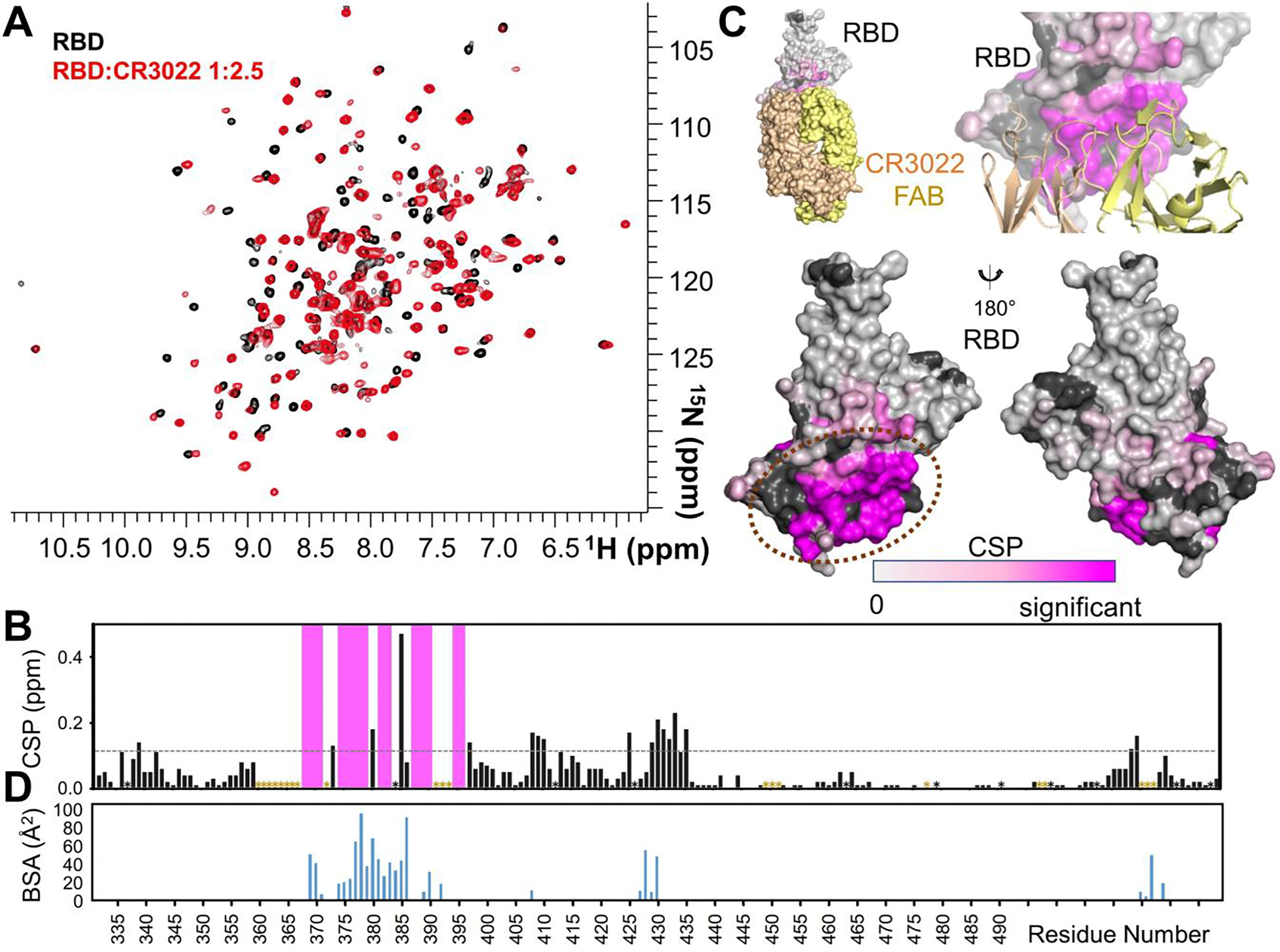
CSP mapping identifies RBD epitopes. **A**. Overlay of the 2D [^1^H,^15^N] TROSY spectrum of the (^2^H,^15^N)-labeled SPIKE SARS2 receptor-binding domain (RBD) in the absence (black) and presence (red; 1:2.25 molar ratio; saturation was reached at a 1:1 molar ratio but the 1:2.25 spectrum, which is shown, has the best S/N ratio due increased recording time) of CR3022 (800 MHz ^1^H Larmor). **B**. CSPs of RBD in the presence of CR3022. Pro residues and residues missing backbone assignment are shown as ‘*’ (black, Pro; yellow, unassigned). The 20 residues that shifted to an extent that they could not be confidently assigned by analyzing nearest peak are shown as pink bars. 19 residues exhibit CSPs greater than 2σ (0.12 ppm, grey dashed line). **C**. RBD CSPs mapped onto the RBD surface with extent of CSP indicated by color (light grey, no observed CSP, to magenta, most significant CSPs). *Upper*, RBD with the CR3022 FAB heavy (yellow) and light (beige) chains shown as either a surface or a cartoon; *lower,* RBD with CSPs mapped onto the surface (proline/unassigned residues in black). Dashed orange ellipse indicates major CR3022 Fab binding interface. **D**. Buried solvent accessible surface area (BSA, buried surface area) of RBD residues when bound to CR3022 (PDBID 6W41).

## References

[1] Polack FP; Thomas SJ; Kitchin N; Absalon J; Gurtman A; Lockhart S; Perez JL; Pérez Marc G; Moreira ED; Zerbini C; Bailey R; Swanson KA; Roychoudhury S; Koury K; Li P; Kalina WV; Cooper D; Frenck RW; Hammitt LL; Türeci Ö; Nell H; Schaefer A; Ünal S; Tresnan DB; Mather S; Dormitzer PR; Şahin U; Jansen KU; Gruber WC; C4591001 Clinical Trial Group. Safety and Efficacy of the BNT162b2 MRNA Covid-19 Vaccine. N Engl J Med 2020, 383 (27), 2603–2615. 10.1056/NEJMoa2034577. 33301246 PMC7745181

[2] Tegally H; Wilkinson E; Giovanetti M; Iranzadeh A; Fonseca V; Giandhari J; Doolabh D; Pillay S; San EJ; Msomi N; Mlisana K; von Gottberg A; Walaza S; Allam M; Ismail A; Mohale T; Glass AJ; Engelbrecht S; Van Zyl G; Preiser W; Petruccione F; Sigal A; Hardie D; Marais G; Hsiao N-Y; Korsman S; Davies M-A; Tyers L; Mudau I; York D; Maslo C; Goedhals D; Abrahams S; Laguda-Akingba O; Alisoltani-Dehkordi A; Godzik A; Wibmer CK; Sewell BT; Lourenço J; Alcantara LCJ; Kosakovsky Pond SL; Weaver S; Martin D; Lessells RJ; Bhiman JN; Williamson C; de Oliveira T Detection of a SARS-CoV-2 Variant of Concern in South Africa. Nature 2021, 592 (7854), 438–443. 10.1038/s41586-021-03402-9. 33690265

[3] Greaney AJ; Starr TN; Gilchuk P; Zost SJ; Binshtein E; Loes AN; Hilton SK; Huddleston J; Eguia R; Crawford KHD; Dingens AS; Nargi RS; Sutton RE; Suryadevara N; Rothlauf PW; Liu Z; Whelan SPJ; Carnahan RH; Crowe JE; Bloom JD Complete Mapping of Mutations to the SARS-CoV-2 Spike Receptor-Binding Domain That Escape Antibody Recognition. Cell Host Microbe 2021, 29 (1), 44–57.e9. 10.1016/j.chom.2020.11.007. 33259788 PMC7676316

[4] Collier DA; De Marco A; Ferreira IATM; Meng B; Datir RP; Walls AC; Kemp SA; Bassi J; Pinto D; Silacci-Fregni C; Bianchi S; Tortorici MA; Bowen J; Culap K; Jaconi S; Cameroni E; Snell G; Pizzuto MS; Pellanda AF; Garzoni C; Riva A; CITIID-NIHR BioResource COVID-19 Collaboration; Elmer A; Kingston N; Graves B; McCoy LE; Smith KGC; Bradley JR; Temperton N; Ceron-Gutierrez L; Barcenas-Morales G; COVID-19 Genomics UK (COG-UK) Consortium; Harvey W; Virgin HW; Lanzavecchia A; Piccoli L; Doffinger R; Wills M; Veesler D; Corti D; Gupta RK. Sensitivity of SARS-CoV-2 B.1.1.7 to MRNA Vaccine-Elicited Antibodies. Nature 2021, 593 (7857), 136–141. 10.1038/s41586-021-03412-7. 33706364 PMC7616976

[5] Walls AC; Park Y-J; Tortorici MA; Wall A; McGuire AT; Veesler D Structure, Function, and Antigenicity of the SARS-CoV-2 Spike Glycoprotein. Cell 2020, 183 (6), 1735. 10.1016/j.cell.2020.11.032. 33306958 PMC7833104

[6] McCallum M; De Marco A; Lempp FA; Tortorici MA; Pinto D; Walls AC; Beltramello M; Chen A; Liu Z; Zatta F; Zepeda S; di Iulio J; Bowen JE; Montiel-Ruiz M; Zhou J; Rosen LE; Bianchi S; Guarino B; Fregni CS; Abdelnabi R; Foo S-YC; Rothlauf PW; Bloyet L-M; Benigni F; Cameroni E; Neyts J; Riva A; Snell G; Telenti A; Whelan SPJ; Virgin HW; Corti D; Pizzuto MS; Veesler D N-Terminal Domain Antigenic Mapping Reveals a Site of Vulnerability for SARS-CoV-2. Cell 2021, 184 (9), 2332–2347.e16. 10.1016/j.cell.2021.03.028. 33761326 PMC7962585

[7] Turoňová B; Sikora M; Schürmann C; Hagen WJH; Welsch S; Blanc FEC; von Bülow S; Gecht M; Bagola K; Hörner C; van Zandbergen G; Landry J; de Azevedo NTD; Mosalaganti S; Schwarz A; Covino R; Mühlebach MD; Hummer G; Krijnse Locker J; Beck M In Situ Structural Analysis of SARS-CoV-2 Spike Reveals Flexibility Mediated by Three Hinges. Science 2020, 370 (6513), 203–208. 10.1126/science.abd5223. 32817270 PMC7665311

[8] Hoffmann M; Kleine-Weber H; Schroeder S; Krüger N; Herrler T; Erichsen S; Schiergens TS; Herrler G; Wu N-H; Nitsche A; Müller MA; Drosten C; Pöhlmann S SARS-CoV-2 Cell Entry Depends on ACE2 and TMPRSS2 and Is Blocked by a Clinically Proven Protease Inhibitor. Cell 2020, 181 (2), 271–280.e8. 10.1016/j.cell.2020.02.052. 32142651 PMC7102627

[9] Wang Q; Zhang Y; Wu L; Niu S; Song C; Zhang Z; Lu G; Qiao C; Hu Y; Yuen K-Y; Wang Q; Zhou H; Yan J; Qi J Structural and Functional Basis of SARS-CoV-2 Entry by Using Human ACE2. Cell 2020, 181 (4), 894–904.e9. 10.1016/j.cell.2020.03.045. 32275855 PMC7144619

[10] Raybould MIJ; Kovaltsuk A; Marks C; Deane CM CoV-AbDab: The Coronavirus Antibody Database. Bioinformatics 2021, 37 (5), 734–735. 10.1093/bioinformatics/btaa739. 32805021 PMC7558925

[11] Yuan M; Wu NC; Zhu X; Lee C-CD; So RTY; Lv H; Mok CKP; Wilson IA A Highly Conserved Cryptic Epitope in the Receptor Binding Domains of SARS-CoV-2 and SARS-CoV. Science 2020, 368 (6491), 630–633. 10.1126/science.abb7269. 32245784 PMC7164391

[12] Yuan M; Liu H; Wu NC; Wilson IA Recognition of the SARS-CoV-2 Receptor Binding Domain by Neutralizing Antibodies. Biochem Biophys Res Commun 2020. 10.1016/j.bbrc.2020.10.012. PMC7547570 33069360

[13] Yuan M; Liu H; Wu NC; Lee C-CD; Zhu X; Zhao F; Huang D; Yu W; Hua Y; Tien H; Rogers TF; Landais E; Sok D; Jardine JG; Burton DR; Wilson IA Structural Basis of a Shared Antibody Response to SARS-CoV-2. Science 2020, 369 (6507), 1119–1123. 10.1126/science.abd2321. 32661058 PMC7402627

[14] Lv H; Wu NC; Tsang OT-Y; Yuan M; Perera RAPM; Leung WS; So RTY; Chan JMC; Yip GK; Chik TSH; Wang Y; Choi CYC; Lin Y; Ng WW; Zhao J; Poon LLM; Peiris JSM; Wilson IA; Mok CKP Cross-Reactive Antibody Response between SARS-CoV-2 and SARS-CoV Infections. Cell Rep 2020, 31 (9), 107725. 10.1016/j.celrep.2020.107725. PMC7231734 32426212

[15] Barnes CO; Jette CA; Abernathy ME; Dam K-MA; Esswein SR; Gristick HB; Malyutin AG; Sharaf NG; Huey-Tubman KE; Lee YE; Robbiani DF; Nussenzweig MC; West AP; Bjorkman PJ SARS-CoV-2 Neutralizing Antibody Structures Inform Therapeutic Strategies. Nature 2020, 588 (7839), 682–687. 10.1038/s41586-020-2852-1. 33045718 PMC8092461

[16] Yuan M; Huang D; Lee C-CD; Wu NC; Jackson AM; Zhu X; Liu H; Peng L; van Gils MJ; Sanders RW; Burton DR; Reincke SM; Prüss H; Kreye J; Nemazee D; Ward AB; Wilson IA Structural and Functional Ramifications of Antigenic Drift in Recent SARS-CoV-2 Variants. 02/17/2021. bioRxiv 10.1101/2021.02.16.430500 (accessed 05/12/2021). PMC8284396 34016740

[17] Pervushin K; Riek R; Wider G; Wüthrich K Attenuated T2 Relaxation by Mutual Cancellation of Dipole-Dipole Coupling and Chemical Shift Anisotropy Indicates an Avenue to NMR Structures of Very Large Biological Macromolecules in Solution. Proc Natl Acad Sci U S A 1997, 94 (23), 12366–12371. 10.1073/pnas.94.23.12366. 9356455 PMC24947

[18] Hussain A; Hasan A; Nejadi Babadaei MM; Bloukh SH; Chowdhury MEH; Sharifi M; Haghighat S; Falahati M Targeting SARS-CoV2 Spike Protein Receptor Binding Domain by Therapeutic Antibodies. Biomed Pharmacother 2020, 130, 110559. 10.1016/j.biopha.2020.110559. 32768882 PMC7395593

[19] ter Meulen J; van den Brink EN; Poon LLM; Marissen WE; Leung CSW; Cox F; Cheung CY; Bakker AQ; Bogaards JA; van Deventer E; Preiser W; Doerr HW; Chow VT; de Kruif J; Peiris JSM; Goudsmit J Human Monoclonal Antibody Combination against SARS Coronavirus: Synergy and Coverage of Escape Mutants. PLoS Med 2006, 3 (7), e237. 10.1371/journal.pmed.0030237. 16796401 PMC1483912

[20] Tian X; Li C; Huang A; Xia S; Lu S; Shi Z; Lu L; Jiang S; Yang Z; Wu Y; Ying T Potent Binding of 2019 Novel Coronavirus Spike Protein by a SARS Coronavirus-Specific Human Monoclonal Antibody. Emerg Microbes Infect 2020, 9 (1), 382–385. 10.1080/22221751.2020.1729069. 32065055 PMC7048180

[21] Morgan WD; Lock MJ; Frenkiel TA; Grainger M; Holder AA Malaria Parasite-Inhibitory Antibody Epitopes on Plasmodium Falciparum Merozoite Surface Protein-1(19) Mapped by TROSY NMR. Mol Biochem Parasitol 2004, 138 (1), 29–36. 10.1016/j.molbiopara.2004.06.014. 15500913

[22] Blech M; Seeliger D; Kistler B; Bauer MMT; Hafner M; Hörer S; Zeeb M; Nar H; Park JE Molecular Structure of Human GM-CSF in Complex with a Disease-Associated Anti-Human GM-CSF Autoantibody and Its Potential Biological Implications. Biochem J 2012, 447 (2), 205–215. 10.1042/BJ20120884. 22839360

