# Supplementary material for: NMR based SARS-CoV-2 Antibody Screening
Source: J Am Chem Soc. Author manuscript; Available in PMC 2025 Nov 18. (PMC12624566; doi:10.1021/jacs.1c03945)
Supplement: Supplementary Material [file NIHMS2108588-supplement-Supplementary_Material.pdf]

Supporting Information

NMR based SARS-CoV-2 Antibody Screening.

Marta V. Schoenle[1,†], Yang Li[2], Meng Yuan[3], Michael W. Clarkson[4], Ian A. Wilson[3,5], Wolfgang Peti[2,*] and Rebecca Page[6,*]

[1]Biochemistry Graduate Program, University of Arizona, Tucson, AZ 85721, USA; [2]Department of Molecular Biology and Biophysics, University of Connecticut Health Center, Farmington, CT 06030, USA; [3]Department of Integrative Structural and Computational Biology, The Scripps Research Institute, La Jolla, CA 92037, USA; [4]Deparment of Chemistry and Biochemistry, University of Arizona, Tucson, AZ 85721 USA; [5]The Skaggs Institute for Chemical Biology, The Scripps Research Institute, La Jolla, CA, 92037, USA; [6]Department of Cell Biology, University of Connecticut Health Center, Farmington, CT 06030, USA

Experimental Protocols and Supplemental Figures

Protein expression. The CR3022 FAB (47.9 kDa) was expressed and purified as previously described[1]. In brief, the plasmids of CR3022 Fab heavy (GenBank: DQ168569.1) and light (GenBank: DQ168570.1) chains were transiently co-transfected into Expi293F cells at a ratio of 2:1 (HC:LC) using ExpiFectamine™ 293 Reagent (Thermo Fisher Scientific) according to the manufacturer's instructions. The supernatant was collected at 7 days post-transfection. The Fab was purified with a CaptureSelect™ CH1-XL Pre-packed Column (Thermo Fisher Scientific) followed by size exclusion chromatography. DNA coding the SARS-CoV-2 RBD (residues 331-528; 22.3 kDa; kindly provided by Dr. Bhattacharya, University of Arizona) in the pET21 was expressed in *E. coli*. Specifically, uniformly (^{1}H,^{15}N)-, (^{2}H,^{15}N)- or (^{2}H,^{15}N,^{13}C)-labeled RBD were expressed in an H_2O/D_2O-based M9 media containing $^{15}NH_4Cl$ (1g/liter) and/or [^{1}H,^{13}C]- or [^{2}H,^{13}C]-D-glucose [4g/liter; CIL (Cambridge Isotope Laboratories)] as the sole carbon and nitrogen sources, respectively. When necessary, cells were gradually transitioned into 100% D_2O-based M9 media in the presence of selective antibiotics at 37°C using rigorous D_2O adaptation. For large scale expression, cells were grown to an OD_{600} (optical density at 600 nm) between 0.8-1.0, induced with 1 mM isopropyl β-D-1-thiogalactopyranoside (IPTG) and the protein allowed to express for 18-20 h at 18°C prior to harvesting at 8,000 *x*g for 15 min at 4°C. Pellets were stored at -80°C.

Inclusion body isolation. Cell pellets were resuspended in solubilization buffer (50 mM Tris-HCl pH 7.5, 25% sucrose, 1 mM EDTA, 10 mM DTT, protease inhibitor cocktail (Roche)) by stirring at room

temperature. Resuspended cells were diluted with 1 volume of lysis buffer (50 mM Tris-HCl pH 7.5, 1% Triton X-100, 100 mM NaCl, 10 mM DTT, 10 mM $MgCl_2$, 50 mM EDTA) and lysed using high-pressure homogenization (Avestin EmulsiFlex C3). The cell lysate was centrifuged at 42,000 *x*g for 1 h at 4°C. The supernatant was discarded and the insoluble fraction containing the inclusion bodies was harvested and stored at -20°C.

RBD refolding. Stored RBD-containing inclusion bodies were washed prior to denaturant solubilization. Specifically, inclusion bodies were thoroughly resuspended in Triton-X wash buffer (50 mM Tris-HCl pH 8.0, 100 mM NaCl, 1 mM EDTA, 1 mM DTT, 0.5% Triton X-100) and pelleted by centrifugation (16,000 *x*g) at 4°C for 30 minutes; this process was repeated three times. The inclusion bodies were then resuspended in wash buffer without Triton-X (50 mM Tris-HCl pH 8.0, 100 mM NaCl, 1 mM EDTA, 1 mM DTT) and pelleted by centrifugation (16,000 *x*g) at 4°C for 30 minutes two more times. The washed inclusion bodies were then solubilized in denaturing buffer (6 M guanidium hydrochloride [GuCl], 20 mM sodium acetate pH 5.2, 20 mM DTT) for 3 h. To refold the RBD, the denatured sample was first dialyzed against a low GuCl buffer (3 M GuHCl, 20 mM sodium acetate pH 5.2, 20 mM DTT) and then refolded using rapid dilution into a redox buffer system by adding the sample dropwise into 400 mL of rapidly stirring refolding buffer (400 mM L-Arginine, 100 mM Tris-HCl pH 7.5, 2 mM EDTA, 5 mM reduced glutathione, 5 mM oxidized glutathione) over a period of 8 hrs. Refolding continued overnight at 4°C with gentle stirring.

RBD purification. The refolded protein sample was concentrated to < 3 mL and purified in a single step using size exclusion chromatography (Superdex 75 16/60, GE Healthcare; equilibrated in NMR buffer: 20 mM MES pH 6.5, 50 mM NaCl). Fractions corresponding to monomeric RBD, as assessed by SDS-PAGE and elution volume, were pooled, concentrated to 10 mg/mL and then flash frozen in liquid nitrogen or used immediately for NMR experiments.

RBD stability measurements. Melting temperature (T_m) measurements that report RBD thermal stability were performed on a Tycho NT.6 (Nanotemper) using standard capillaries (10 μL) and a 30 °C/min ramp (from 35 to 95 °C). The data was evaluated using the Tycho NT.6 software version 1.1.5.668.

RBD sequence-specific backbone assignment. All sequence-specific backbone assignment and $^{15}N\{^{1}H\}$-NOE data collections were performed at 298 K on a Bruker Avance NEO 600 MHz spectrometer equipped with TCI HCN z-gradient cryoprobe. NMR samples of (^{2}H,^{15}N)- or (^{2}H,^{15}N,^{13}C)-labeled RBD (450 μM) were prepared in NMR buffer (20 mM MES pH 6.5, 50 mM NaCl) in 90% H_2O/10% D_2O and used immediately for data acquisition. The following experiments were recorded: 2D [^{1}H,^{15}N] TROSY, 3D HNCA, 3D HNCACB, 3D HN(CO)CA, 2D HN(CO)CACB and 3D ^{15}N-filtered [^{1}H-^{1}H] NOESY (for all 3D spectra TROSY versions were used for data acquisition[2]). The NMR spectra were processed with Topspin 4.05 (Bruker) and analyzed using the CARA software package (http://cara.nmr.ch). $^{15}N\{^{1}H\}$-NOE-TROSY data was acquired (pseudo-3D interleaved) using a 300 μM (^{2}H,^{15}N)-labeled RBD sample (90% H_2O/10% D_2O). A saturation period of 5 s and a relaxation delay of 4 s was used for a total acquisition time of 105 hrs. Intensities were extracted using CCPN[3] and ratios and errors were calculated using Microsoft Excel.

CR3022:RBD interaction studies. For NMR interaction experiments, CR3022 was dialyzed against NMR buffer for ~16 hrs at 4°C. CR3022 was added to (^{2}H,^{15}N)-labeled RBD (40 μM), incubated for 30 minutes and a series of 2D [^{1}H,^{15}N] TROSY spectra were recorded with samples have RBD:CR3022 ratios of 1:1, 1:2 and 1:2.5. Data were recorded on a Bruker Avance NEO 800 MHz spectrometer equipped with TCI HCN z-gradient cryoprobe at 298 K. Cross-peaks that barely shifted were assigned by overlaying with the CR3022 free RDB spectrum, and for all peaks that showed significant chemical shift perturbations (CSPs, $\Delta\delta$), nearest neighbor chemical shift difference analysis was performed. CSPs were calculated using:

$$\Delta\delta(ppm) = \sqrt{(\Delta\delta_H)^2 + \left(\frac{\Delta\delta_N}{5}\right)^2}$$

Supporting References

(1) Yuan, M.; Wu, N. C.; Zhu, X.; Lee, C.-C. D.; So, R. T. Y.; Lv, H.; Mok, C. K. P.; Wilson, I. A. A Highly Conserved Cryptic Epitope in the Receptor Binding Domains of SARS-CoV-2 and SARS-CoV. Science 2020, 368 (6491), 630–633. https://doi.org/10.1126/science.abb7269.

(2) Pervushin, K.; Riek, R.; Wider, G.; Wüthrich, K. Attenuated T2 Relaxation by Mutual Cancellation of Dipole-Dipole Coupling and Chemical Shift Anisotropy Indicates an Avenue to NMR Structures of Very Large Biological Macromolecules in Solution. Proc Natl Acad Sci U S A 1997, 94 (23), 12366–12371. https://doi.org/10.1073/pnas.94.23.12366.

(3) Skinner, S. P.; Fogh, R. H.; Boucher, W.; Ragan, T. J.; Mureddu, L. G.; Vuister, G. W. CcpNmr AnalysisAssign: A Flexible Platform for Integrated NMR Analysis. J Biomol NMR 2016, 66 (2), 111–124. https://doi.org/10.1007/s10858-016-0060-y.

Figure S1

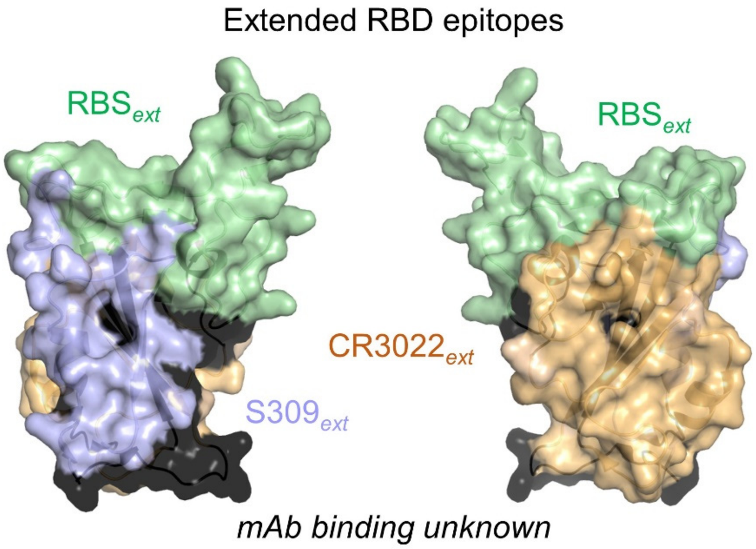

Figure S1. **Extended RBD Epitopes**. Of the 1150 mAb isolated that target the RBD, the RBD binding epitopes of 116 have been determined to high resolution (X-ray crystallography and/or cryo-EM). The extended epitopes of the RBS (green), CR3022 (orange) and S309 (lavender) are shown. Black are those residues that, thus far, have not been identified as part of an RBD:mAb or RBD:Fab interface (i.e., they do not experience any loss in solvent accessible surface area upon mAb/Fab binding). Our data suggest that these epitopes can be further refined into sub-epitopes using NMR CSP data. Further, adding NMR CSP experiments to crystallographic and cryo-EM efforts will rapidly increase our ability to molecular characterize RBD epitopes, accelerating vaccine and antibody research.
